# Photoresponsive tetracoordinate arylboron smart molecules: Strategies for molecular design and photoresponse mechanisms

**DOI:** 10.1002/smo.20240041

**Published:** 2024-11-11

**Authors:** Jinjin Wang, Mengzhen Li, Haoyu Gao, Lixia Xie, Xin Zheng, Guoxing Liu, Tianjing Wu, Lu Lin, Lijie Liu

**Affiliations:** ^1^ College of Science Henan Agricultural University Zhengzhou Henan China; ^2^ School of Chemistry Xiangtan University Xiangtan China

**Keywords:** organoboron photochemistry, organoboron photochromism, organoboron photoisomerization, organoboron photoresponsive materials

## Abstract

Photoresponsive smart materials, which exhibit swift or instantaneous responses to external light stimuli, are pivotal for advancing the development of novel smart devices. Among these materials, photoresponsive tetracoordinate arylboron compounds emerge as prominent molecular systems, owing to their captivating photochemical mechanisms and photophysical transformations. In recent years, these molecules have experienced notable progress, leading to the emergence of numerous organic boron photoresponsive molecular systems with innovative structures and exceptional performance. In this comprehensive review, we present a thorough examination of the latest advancements in the field, systematically elucidating the design strategies and structure‐activity relationships of these molecules. Furthermore, we delve into the photoresponse mechanisms of various molecules and summarize their unique characteristics. Ultimately, we analyze the challenges, opportunities, and prospects encountered in this exciting field of research.

## Graphical abstract

Photoresponsive tetracoordinate arylboron compounds constitute significant molecular systems in the fabrication of photoresponsive smart materials. In this review, we present a comprehensive overview of the recent advancements in this domain, providing a systematic exposition of the molecular structure design strategies, structure‐activity relationships, and photoresponse mechanisms of these compounds.

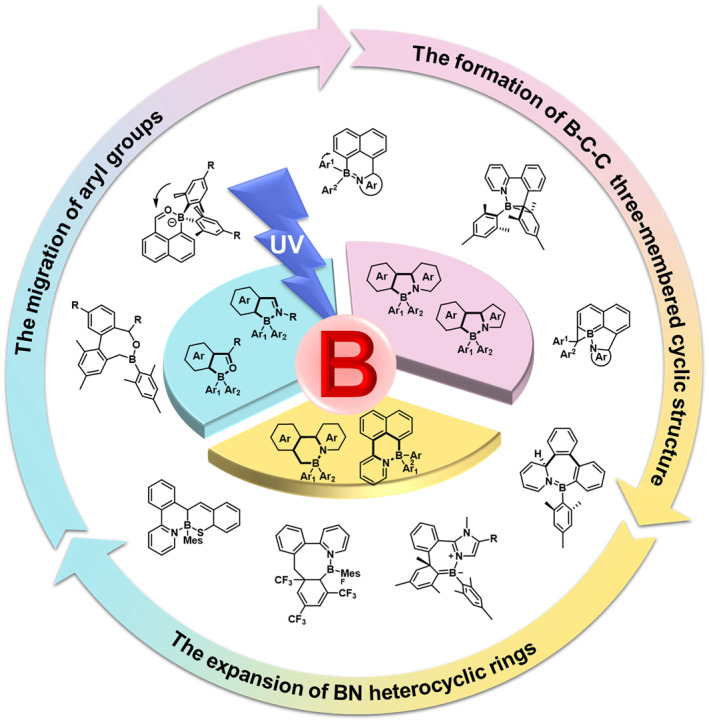

## 1 INTRODUCTION

Stimuli‐responsive materials, a prominent class of smart materials, have garnered significant attention and have been widely applied in biosensing, information storage, and wearable devices.[^1^, ^2^, ^3^, ^4^, ^5^, ^6^] These materials have become a focal point of research in chemistry, materials science, and biology. Among them, photoresponsive smart materials are particularly noteworthy for their capability to undergo rapid or immediate changes in their chemical, physical, and electronic properties, such as color, luminescence, conductivity and refractive index when exposed to external light stimuli.[^7^, ^8^, ^9^] This remarkable property endows photoresponsive materials with a vast potential for applications in optical storage devices, molecular switches, photochromic glasses, and smart windows.[^10^, ^11^, ^12^]

**FIGURE 1:**
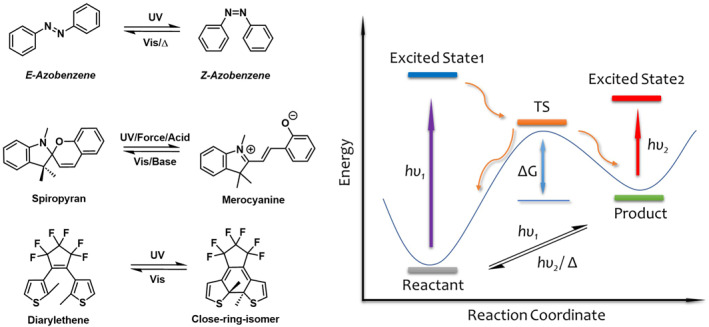
Example of azobenzene, spiropyran, and diarylethene for photoresponsive systems[^13^, ^16^, ^17^, ^18^] (left), dynamics mechanism of photoresponsive isomerization[^7^, ^19^] (right).

Traditional photoresponsive materials encompass both inorganic and organic types. Among these, organic photoresponsive materials have garnered extensive attention and research due to their tunable structures and diversity, which allow for a broader range of applications.[^13^, ^14^, ^15^] This versatility has made them a long‐standing focus of research within the scientific community. Prominent organic photoresponsive molecular systems currently under extensive investigation include those based on cis‐trans isomerization mechanisms such as alkenes and azobenzenes, spiropyran molecules, and diarylethene systems utilizing photoinduced ring‐opening/closing mechanisms (Figure 1).[^13^, ^16^, ^17^, ^18^] These molecular systems exhibit excellent photoresponsive properties and their underlying response mechanisms have been thoroughly explored. However, there remains a scarcity of new and reliable photoresponsive molecular systems, highlighting the need for further exploration and development in this field.

Organoboron compounds, owing to the empty p‐orbitals of the boron (B) atom, can form strong coordination bonds with atoms such as nitrogen (N), phosphorus (P), oxygen (O), and sulfur (S), giving rise to a diverse array of molecule systems.[^20^, ^21^, ^22^, ^23^, ^24^, ^25^, ^26^, ^27^, ^28^] Notably, these compounds, especially those with tetracoordinated organic boron, exhibit unique photoresponsive properties characterized by changes in absorption and emission spectra. They represent a novel and superior class of photoresponsive molecular systems.[^29^, ^30^, ^31^] In 2005, Kawashima et al.^[32]^ documented the reversible photoresponse behavior observed in a class of azobenzene tetracoordinated organic boron compounds. This behavior is attributed to the Z‐E configuration inversion mechanism induced by light exposure. Over the years, numerous organoboron photoresponsive materials have emerged through sustained research efforts.

**FIGURE 2:**
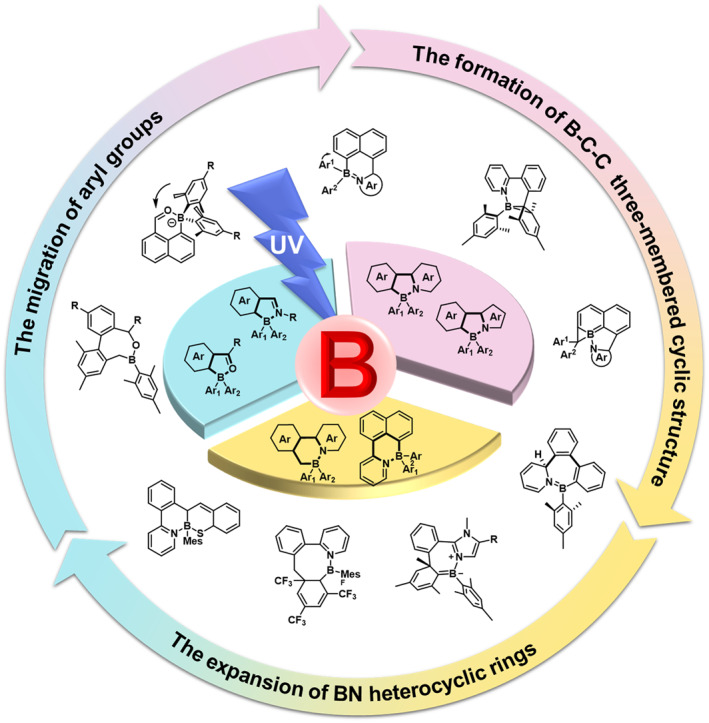
Molecular systems of photoresponsive tetracoordinate arylboron compounds.[^7^, ^19^, ^29^, ^35^, ^36^]

Among various organic boron molecular systems, arylboron compounds are distinguished by their exceptional stability in water and oxygen. This stability enables their widespread application in semiconductor materials, thermally activated delayed fluorescence (TADF) materials, and bioimaging materials.[^14^, ^19^, ^33^, ^34^] Additionally, these compounds demonstrate a rich photochemical nature and notable versatility and tunability in photoresponsive colors changes, establishing a comprehensive and systematic organoboron photoresponsive molecular system.[^29^, ^35^, ^36^] This article is dedicated to providing a systematic review of the development process, molecular structure design strategies, structure‐activity relationships, photoresponse mechanisms, and applications of photoresponsive arylboron compounds (Figure 2). Furthermore, it strives to explore the development trends and future prospects of these fascinating molecules.

## 2 STRUCTURAL CHARACTERISTICS OF PHOTORESPONSIVE TETRACOORDINATED ARYLBORON COMPOUNDS

The photoresponsive tetracoordinated arylboron compounds typically consist of an L‐BAr_2_ system. Here, L represents the primary ligand, which is formed by the connection of two aromatic rings with coordination sites via a single bond (such as 2‐biphenyl‐pyridine (PPy) or 2‐naphthyl‐pyridine). Ar_2_ stands for two aromatic rings possessing a certain degree of steric hindrance (including mesityl (Mes), phenyl, or thienyl). These aromatic rings serve to protect the B atom, ensuring its stability under ambient conditions. Generally, the photoisomerization behavior of these molecular systems can be adjusted by modifying the structures of both L and Ar.

A classic example is when L is a 2‐phenylpyridine analog (such as **PPyBMes**_**2**_). Upon irradiation, it undergoes dearomatization of the mesityl groups, resulting in the formation of a B‐C‐C three‐membered cyclic structure.^[37]^ This transformation is accompanied by a notable darkening of color and the appearance of a broad absorption peak in the long‐wavelength region of the absorption spectrum, with a concomitant gradual decrease in fluorescence.

**FIGURE 3:**
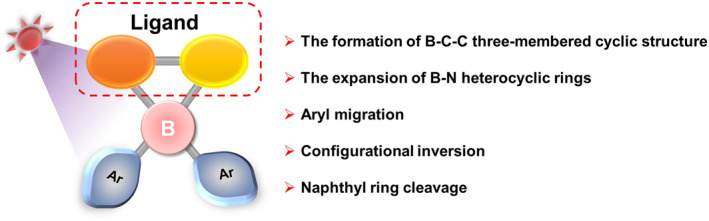
Structural characteristics of photoresponsive tetracoordinated arylboron compounds.[^7^, ^19^, ^35^, ^36^]

Moreover, by adjusting the structure of the aromatic rings in L and Ar, it is possible to achieve new isomerization behaviors after the formation of the B‐C‐C three‐membered cyclic structure, leading to the formation of novel BN‐containing six‐membered, seven‐membered, or even eight‐membered ring structures (Figure 3).[^38^, ^39^, ^40^]

Additionally, apart from the dearomatization mechanism leading to the formation of the B‐C‐C three‐membered cyclic structure, tetracoordinate arylboron compounds also exhibit other isomerization behaviors, including aryl migration, configurational inversion, and even naphthyl ring cleavage. The study of these mechanisms provides a solid theoretical basis for the molecular design of photoresponsive tetracoordinate arylboron compounds.

Next, we will embark on an exploration of the impact of diverse molecular structures on photoisomerization behavior, grounded in their distinctive characteristics. This investigation will involve a comprehensive introduction of photoresponsive tetracoordinated arylboron compounds, categorized according to their unique photoisomerization mechanisms and addressed from multiple perspectives.

## 3 THE PHOTORESPONSIVE BEHAVIOR OF N‐C CHELATED ARYLBORON COMPOUNDS: THE MECHANISM OF MESITYL GROUP DEAROMATIZATION AND THE FORMATION OF B‐C‐C THREE‐MEMBERED CYCLIC STRUCTURE

Firstly, we focus on the photoresponsive behavior of L‐BAr_2_‐type derivatives, specifically exploring the mechanism behind the dearomatization of the mesityl group and the consequent formation of a B‐C‐C three‐membered cyclic structure. Such transformations are the key to understanding the unique photoresponsive properties of these compounds.

### 3.1 The regulation of photoresponsive behavior in tetracoordinate arylboron compounds via modification of the primary ligand (L) structure

#### 3.1.1 The regulatory effect of modifications in phenylpyridine (PPy)‐based primary ligands

**FIGURE 4:**
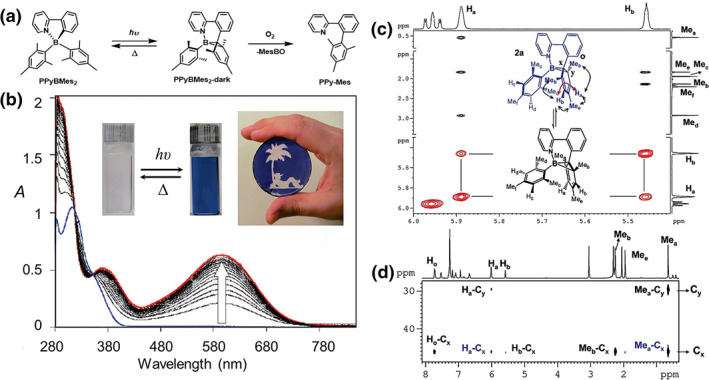
(a) The isomerization of **PPyBMes**_**2**_ and the reaction of **PPyBMes**_**2**_ **‐dark** with O_2_. (b) UV‐vis spectral change of **PPyBMes**_**2**_ upon irradiation by UV light (365 nm) in toluene. (c) Portions of the ^1^H NOESY spectrum of **PPyBMes**_**2**_ at 298 K in C_6_D_6_ showing the exchange cross‐peak H_a_↔H_b_ (red) and their NOE cross‐peaks (black) with methyls. (d) A partial HMBC spectrum of **PPyBMes**_**2**_ in C_6_D_6_ at 283 K showing H_a_, Me_a_, and C_*x*_ correlation peaks. Reproduced with permission.^[35]^ Copyright 2008, American Chemical Society.

In 2008, Wang et al.^[35]^ first reported the unique reversible photo‐thermal color switching behavior of tetracoordinate boron compounds based on PPy ligands. Under 365 nm UV light irradiation, compound **PPyBMes**_**2**_ undergoes a color change from colorless to deep blue, accompanied by fluorescence quenching (Figure 4b). The absence of a significant ESR signal in the deep blue solution at both 298 and 77 K excludes the involvement of radicals in the observed color change.

The intense and dark colors of **PPyBMes**_**2**_ **‐dark** can be attributed to a low energy charge transfer transition from the B‐cyclohexadienyl moiety to the BMes_2_‐Py‐Ph and Py‐Ph moieties, respectively. This was confirmed by ^1^H NMR and X‐ray single‐crystal analysis, which demonstrated that under 365 nm UV light irradiation, the B‐C bond cleaves and bonds with the benzene ring on mesityl, disrupting the aromaticity of the mesityl group and forming a distinctive B‐C‐C three‐membered cyclic structure. Upon heating in a nitrogen environment, **PPyBMes**_**2**_ **‐dark** can revert to its original structure. In the presence of oxygen, however, the unreacted BMes_2_ is eliminated, resulting in the formation of **PPy‐Mes**. Although the high sensitivity of **PPyBMes**_**2**_ to oxygen under light exposure may limit the use of such materials in certain optoelectronic devices, the facile and reversible B‐C bond formation and cleavage process offers potential applications in the synthesis of organoboron compounds. Following this study, the development of photoisomeric molecules based on tetracoordinated arylboron compounds has gradually progressed.

**FIGURE 5:**
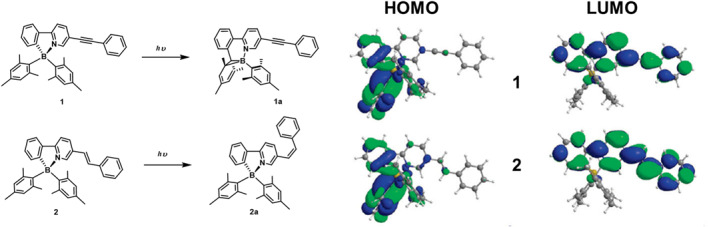
Photoisomerization process of **1** and **2,** HOMO and LUMO orbitals of **1** and **2** with an isocontour value of 0.02 for all surfaces. Reproduced with permission.^[41]^ Copyright 2009, American Chemical Society.

In 2009, Baik et al.^[41]^ investigated the effects of incorporating alkenes and alkynes into the **PPyBMes**_**2**_ system on its photoisomerization process. The alkyne‐based compound **1** exhibited the same photochemical isomerization behavior as **PPyBMes**_**2**_ (Figure 5). However, its quantum yield for photoconversion was only 0.33, significantly lower than the 0.88 observed for **PPyBMes**_**2**_. This reduction is attributed to the expanded *π*‐conjugation introduced by the alkyne group, which stabilizes the excited state and reduces photoreactivity. In contrast, the alkene‐based compound **2** did not show a significant color change under UV irradiation, but its trans isomer readily underwent cis isomerization, with a conversion rate of 70%. TD‐DFT calculations revealed that under UV irradiation, compound **2** forms an excited‐state single bond that facilitates trans‐to‐cis isomerization providing an alternative energy dissipation pathway for the excited state of **PPyBMes**_**2**_. However, for alkyne compounds, although the triple bond is weakened, it can not undergo configurational inversion or isomerization like the alkene compounds. This suggests that the introduction of a trans‐cis isomerization pathway is an effective strategy for stabilizing photochemically unstable chromophores in π‐conjugated systems.

**FIGURE 6:**
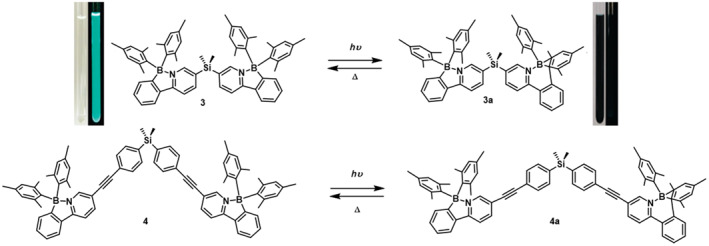
Photoisomerization process of **3** and **4**. Reproduced with permission.^[42]^ Copyright 2010, American Chemical Society.

In 2010, Murphy et al.^[42]^ investigated the influence of steric hindrance on the photoisomerization quantum efficiency of **PPyBMes**_**2**_ units. By bridging two **PPyBMes**_**2**_ units with a silicon linker, **3** exhibited structural transformation under UV light, where only one **PPyBMes**_**2**_ unit underwent a change from colorless to deep purple, accompanied by quenched green fluorescence (Figure 6). This process was reversible upon heating. The phenomenon of single‐unit isomerization can be attributed to intramolecular energy transfer based on the Kasha principle, where energy from non‐isomerizable groups dissipates through spatial transfer to the lower energy absorption band of the isomerizable group, thereby preventing isomerization of the second group. Connecting two photo‐switchable boron‐based units via a silicon bridge significantly enhances the photoisomerization quantum efficiency in spatial non‐crowded systems. In the photoisomerization process of the dual **PPyBMes**_**2**_ unit compound, intra‐molecular energy transfer and spatial factors play crucial roles.

**FIGURE 7:**
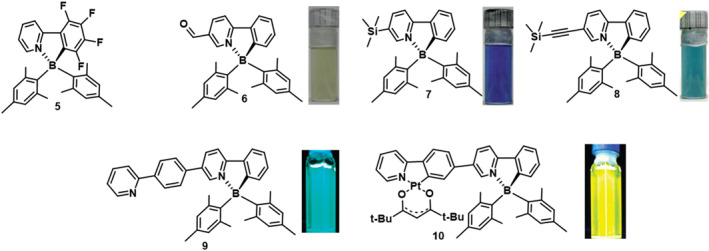
Molecular structures of **5‐10**, fluorescent or daylight photo after photoisomerization. Reproduced with permission.^[43]^ Copyright 2010, John Wiley and Sons. Reproduced with permission.^[37]^ Copyright 2011, American Chemical Society.

In 2010, Amarne et al.^[43]^ conducted a series of modifications on **PPyBMes**_**2**_ to investigate the effects of different donor and acceptor groups on its photoresponsive behavior (Figure 7). The study revealed that compounds **6** ‐ **8** exhibited photochromism under 365 nm UV light, transitioning from colorless or pale yellow to deep blue or green. Conversely, compound **5** decomposed under 350 nm irradiation. The findings indicate that incorporating electron‐donating groups into the ligand destabilizes the excited state, accelerating the photoisomerization rate. In contrast, introducing electron‐withdrawing groups slows down the photoisomerization process or leads to irreversible photodecomposition, hindering the development of stable and reversible photoswitch systems.

In 2011, Rao et al.^[37]^ conjugated Pt phosphorescent moieties with **PPyBMes**_**2**_ and investigated the impact of covalently bound transition metal ions on the photochromic properties of these boron compounds. Upon irradiation at 365 nm, the color of **9** solution changed to deep green, and the photoisomerization from **9** to **9a** was completely thermally reversible. **10** underwent photoisomerization in a similar manner to **9** but with significantly lower quantum efficiency. The low photoisomerization quantum efficiency of **10** can be attributed to their low‐lying ^3^LC state, effective spin‐orbit coupling induced by Pt(II) chelation, and intersystem crossing between singlet and triplet states, which serve as efficient deactivation pathways. Enhancing charge transfer transitions from mesityl groups to the chelate and disrupting *π*‐π* transitions of the chelate backbone are critical factors in achieving a novel and efficient photochromic system based on **PPyBMes**_**2**_.

**FIGURE 8:**
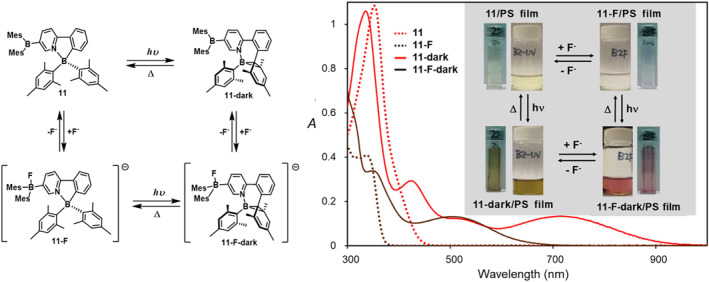
Four‐state reversible switching of **11**: Molecular structure and UV−vis spectra of the four diboron species involved in the four‐state color switching of **11.** Reproduced with permission.^[36]^ Copyright 2016, American Chemical Society.

Triarylboron compounds can coordinate with fluoride ions, thereby inducing color changes. Introducing tricoordinate boron compounds into **PPyBMes**_**2**_ may result in additional color variations. In 2016, Mellerup et al.^[36]^ combined the BMes_2_ moiety with **PPyBMes**_**2**_, synthesizing a novel diboron compound **11**. Upon reaction with TBAF, the fluorescence color of **11** changed from green to deep blue. The addition of F⁻ significantly altered the color of the dark isomer of **11**. The structure of the **11‐F⁻** dark isomer generated by F⁻ is identical to the product obtained from direct photoisomerization of **11**. **11** can undergo a four‐state reversible color transition under the stimuli of light, heat, and fluoride ions (Figure 8). Therefore, the diboron system not only facilitates color tuning and switching in this photochromic system but also enables visual fluoride sensing through changes in color or fluorescence emission.

**FIGURE 9:**
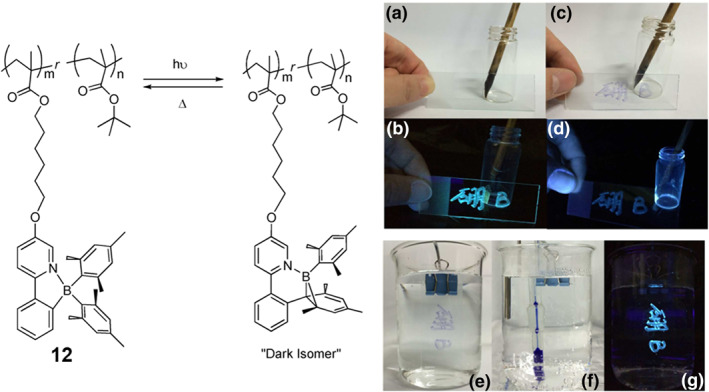
(a) Photochromic polymers/random copolymers based on the **PPyBMes**_**2**_ unit (left). The writing with a Chinese brush and **12** polymer as the ink on glass (right). Reproduced with permission.^[44]^ Copyright 2011, American Chemical Society.

When **PPyBMes**_**2**_ is incorporated into polymers, it can also undergo photoisomerization, enabling the use of this compound as a polymer‐based photochromic material in the solid state. For example, polymer **12** was synthesized using a mixture of a methacrylate‐derived photochromic unit (BHMA) and tert‐butyl methacrylate via atom transfer radical polymerization (ATRP). The polymer exhibited absorption in the ultraviolet region (*λ*_abs_ = 358 nm) and displayed intense blue emission (*λ*_em_ = 476 nm, Φ_F_ = 43%). Upon irradiation, a deep blue color (*λ*_abs_ = 580 nm) was observed in solution, and a reversible photochromic process was demonstrated in the film state, unaffected by water and oxygen (Figure 9). This photochromic polymer can be directly applied as a surface coating or used as ink for writing, further expanding the potential applications of these types of boron compounds.

#### 3.1.2 The regulatory effect of modifications in heterocyclic based primary ligands

**FIGURE 10:**
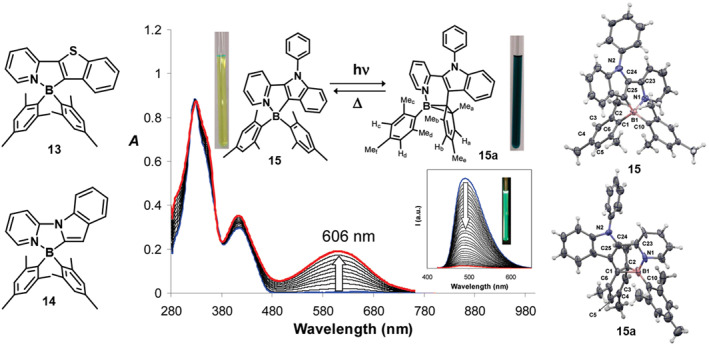
Molecular structure of **13‐15**, UV‐vis spectral change of **15** in toluene upon irradiation by UV light, and Crystal structure of compounds **15** and **15a.** Reproduced with permission.^[45]^ Copyright 2011, American Chemical Society.

To further investigate the mechanism of photoisomerization, Hazem Amarne and colleagues^[43]^ explored the influence of five‐membered heterocyclic ligands on this process (Figure 10). These ligands specifically feature benzothiophene **13** and indole **14** as the aryl rings, where the boron atom is bonded through the C atom of the heterocyclic ring without altering their photochemical properties. Under UV irradiation at 365 nm, compound **13** changes from being colorless to deep green. However, **14** decomposes when exposed to 350 nm irradiation, likely due to the instability of the indole‐pyridine chelate.

Subsequently, by connecting the phenyl ring with indole, the conjugation system of the ligand was expanded, resulting in compound **15**,^[45]^ which allows for the stabilization of its dark isomer molecular structure (Figure 10). In comparison to **PPyBMes**_**2**_, compound **15** displays a significantly lower isomerization yield of just 0.09. This decrease is primarily attributed to steric hindrance caused by the phenyl of the indole group, which obstructs photoisomerization. In its solid state, **15a** exhibits enhanced oxygen stability. This improvement is potentially linked to stronger intermolecular *π*‐π interactions and tight lattice packing facilitated by the N‐phenyl ring.

**FIGURE 11:**
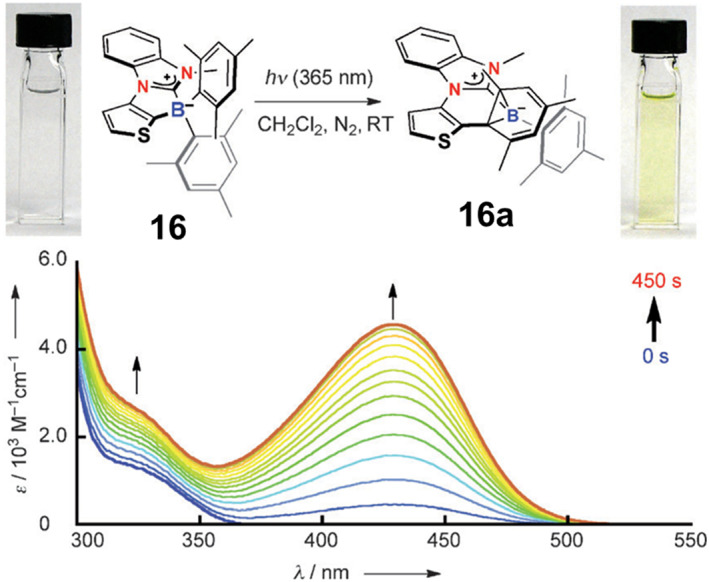
Photoreaction of **16** with a picture of the solution before and after irradiation by UV light, and absorption spectral change of a solution of **16** in CH_2_Cl_2_ upon light irradiation. Reproduced with permission.^[46]^ Copyright 2012, Royal society chemistry.

In 2012, Yamaguchi et al.^[46]^ reported N‐Heterocyclic carbene (NHC)‐borane‐substituted thiophene **16**. Under 365 nm UV irradiation, it changes from colorless to deep yellow as **16a**, with a new absorption peak emerging at 429 nm (Figure 11). This process also involves the formation of a B‐C‐C three‐membered cyclic structure. The color change is attributed to an ICT transition from the HOMO on the borabicyclo[4.1.0]heptadiene skeleton to the LUMO on the NHC moiety. However, **16a** demonstrates good thermal stability and cannot revert to **16** upon heating, owing to the stronger coordination ability between the NHC and the borabicyclo[4.1.0]heptadiene skeleton.

**FIGURE 12:**
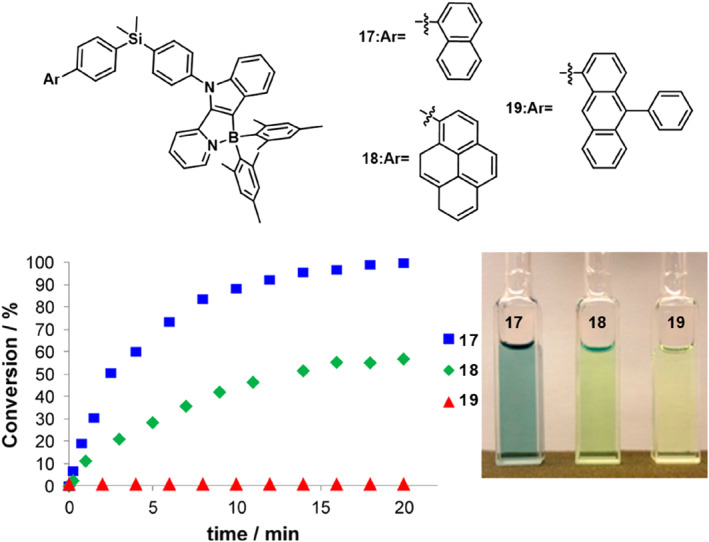
Left: Conversion of **17‐19** to their dark isomers over a 20 min period under irradiation at 419 nm. Right: Photos of **17‐19,** respectively, after 10 min of irradiation. Reproduced with permission.^[47]^ Copyright 2012, Royal society chemistry.

Based on compound **15**, Hudson et al.^[47]^ explored the influence of aromatic ring size on the photoisomerization yield of this series of compounds (Figure 12). They synthesized a range of photoisomeric compounds **17**‐**19** using pyridine‐benzopyrrole derivatives as ligands. Under 419 nm light, compounds **17** and **19** form dark isomers. However, upon prolonged illumination, only a small number of compound **19** converts to its dark isomer. The thermal reversal of compound **17** is relatively slow, while compounds **18** and **19** show no significant transformation upon heating. Theoretical calculations suggest that the low triplet state of the acceptor may be close to the activation energy of thermal reversal, thus inhibiting the thermal conversion process of the dark isomers of compounds **18** and **19**.

### 3.2 The regulation of photoresponsive behavior in tetracoordinate arylboron compounds via modification of the aryl (Ar) connected to boron

**FIGURE 13:**
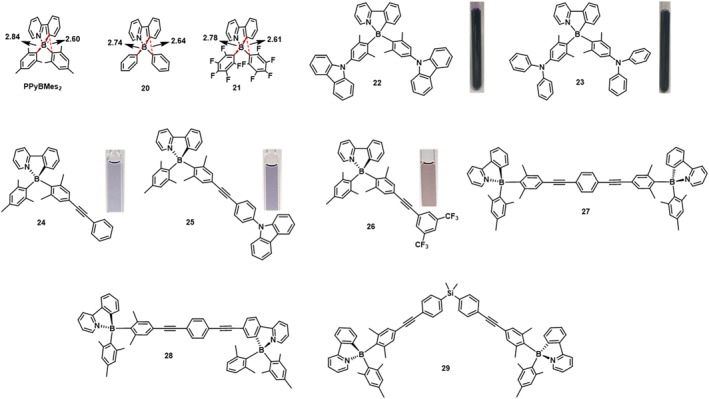
Molecular structures of **20**‐**29**, and photos after irradiation.[^43^, ^48^, ^49^] Reproduced with permission.^[43]^ Copyright 2010, John Wiley and Sons. Reproduced with permission.^[48]^ Copyright 2016, John Wiley and Sons. Reproduced with permission.^[49]^ Copyright 2018, American Chemical Society.

In 2010, Amarne et al.^[43]^ synthesized a series of tetracoordinate N,C‐chelate boron compounds, including diphenyl compounds **20** and **21**, which remained stable under UV irradiation at 350 or 310 nm (Figure 13). Compared to **PPyBMes**_**2**_, the two phenyl rings in compound **20** have significantly shorter C‐B bonds. The energy charge transfer from mesityl→PPy in **PPyBMes**_**2**_ was lower than that of phenyl→PPy in **20** due to the weaker B‐Mes bond and the electron‐donating effect of the methyl groups. Compound **21**, lacking additional hydrogen atoms on the boron‐bound aryl groups, did not undergo photoisomerization. These results indicate that the steric hindrance of substituents on the boron atom is a critical factor in determining the feasibility of photochemical reactions.

In 2016, Mellerup et al.^[48]^ introduced different types of amine‐based electron‐donating groups to the boron aryl moiety, investigating their photoisomerization behavior (Figure 13). Under a 350 nm UV lamp, compounds **22** and **23** exhibited photochromic behavior similar to **PPyBMes**_**2**_, changing from colorless to purple (**22**) and from yellow to deep blue (**23**), respectively. By modifying the HOMO energy of the electron‐donating aryl unit of the boron compound, the color of the dark isomer can be controlled. The isomerization rates of **22** and **23** relative to **PPyBMes**_**2**_ are 0.64 and 0.10, respectively. The lower photoisomerization efficiency of **23** may be attributed to the increased non‐radiative rate constant caused by the additional rotational freedom of the triphenylamine.

To further investigate the impact of extending the conjugation system of aryl substituents on boron on photoisomerization, asymmetric boron compounds **24‐29**,^[49]^ containing a mesityl group and an alkynyl derivative, were synthesized (Figure 13). Among them, monoboron compound **24** undergoes a color change from colorless to deep purple under 350 nm irradiation, and its dark isomer can revert to the original isomer upon heating. Linear compounds **27** and **28**, on the other hand, exhibit stability towards both 300 and 350 nm irradiation, lacking the required charge transfer (CT) from *π*‐Ar (HOMO) to π*‐PPy as the predominant component of their S1 state, thus failing to undergo photoreaction. Compound **29**, interestingly, shows isomerization of only one chromophore upon irradiation, possibly attributed to the greater extent of *π*‐conjugation in the aryl group involved in the isomerization process.

## 4 THE PHOTORESPONSIVE BEHAVIOR OF N‐C CHELATED ARYLBORON COMPOUNDS: FROM B‐C‐C THREE‐MEMBERED CYCLIC STRUCTURE TO THE EXPANSION OF BN HETEROCYCLIC RINGS

The photoresponsive formation of the B‐C‐C three‐membered cyclic structure in many N‐C chelated arylboron compounds is unstable, often resulting in the departure of the arylboron group when exposed to air. However, there are certain molecules that undergo ring expansion upon further irradiation or heating after photochemical conversion into the B‐C‐C cyclic structure, leading to the formation of novel BN heterocyclic rings. To gain a deeper understanding of this transformation, we shall explore the structures and reaction mechanisms of these photoresponsive molecules.

### 4.1 The regulation of photoresponsive behavior in tetracoordinate arylboron compounds via modification of the primary ligand (L) structure

**FIGURE 14:**
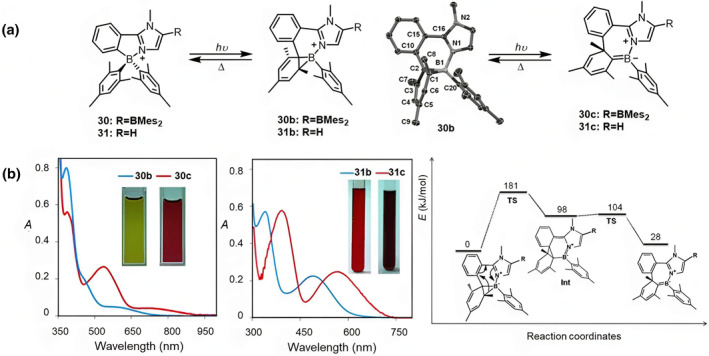
(a) Photoisomerization of compounds **30** and **31** and Crystal structure of **30b** (b) UV spectra of isomers **b** and **c** Reproduced with permission.^[50]^ Copyright 2014, John Wiley and Sons.

Upon the introduction of imidazole into the chelating ligand (L = phenylimidazole), both compounds **30** and **31** initially form a tricyclic ring under 350 nm irradiation, exhibiting color changes to olive green and red, respectively (Figure 14).^[50]^ Upon further irradiation, these compounds undergo a second isomerization process, resulting in color transitions to red (**30b**) and red‐brown (**31b**). At 80°C, compounds **30c** and **31c** can be thermally reduced to **30** and **31b**, respectively. At a higher temperature (110°C), isomer **b** can fully revert to isomer **a**, completing a reversible dual‐process molecular isomerization. Theoretical calculations were performed for the transition from **31c** to **31b**. The conversion of **31c** to the intermediate (**Int**) involves an electrocyclic rearrangement, while the transition from Int to **31b** entails a rearrangement of the cyclohexadienyl unit. The calculated transition states and intermediates of **31b** and **31c** involve changes in B‐N bond order and dearomatization of the imidazole ring. The *σ*‐donating strength and aromaticity of the heterocyclic ring bound to the B atom are key factors determining the specific isomerization pathway among competing routes.

**FIGURE 15:**
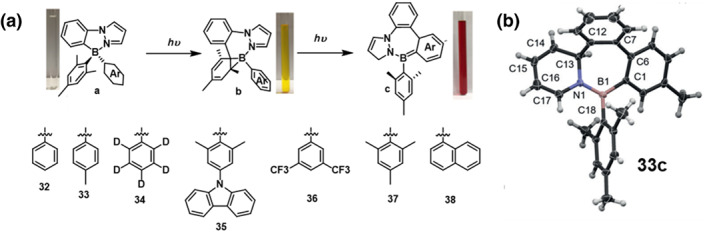
Molecular structure of **32‐38** and Crystal structure of **33c**. Reproduced with permission.^[51]^ Copyright 2018, American chemical society.

In 2018, Li et al.^[51]^ synthesized a series of phenyl‐pyrazole based organoboron compounds. Among them, compound **32** underwent a two‐stage color change under a 300 nm UV lamp: from colorless to yellow, and then to deep orange (Figure 15). Compound **32** experiences the same regioselective photoisomerization as the B(PPy)(Mes)(Ar) molecule, where the B‐C‐C three‐membered cyclic structure is only formed on the smaller benzene ring. Compounds **33**‐**38** undergo initial regioselective photoisomerization into their respective borane isomers “**a**”. Irradiation at 300 nm induces all compounds to form a broad CT (HOMO→LUMO) absorption band between 370 and 440 nm, giving each dark isomer a light yellow appearance. Due to significant steric hindrance, prolonged irradiation of compounds **37** and **38** does not lead to a structural transition from **b** to **c**. This is the first observed reaction pathway in chiral N, C‐chelated arylboron compounds, illustrating that multiple excited state pathways can be accessed by modifying their electronic structures.

**FIGURE 16:**
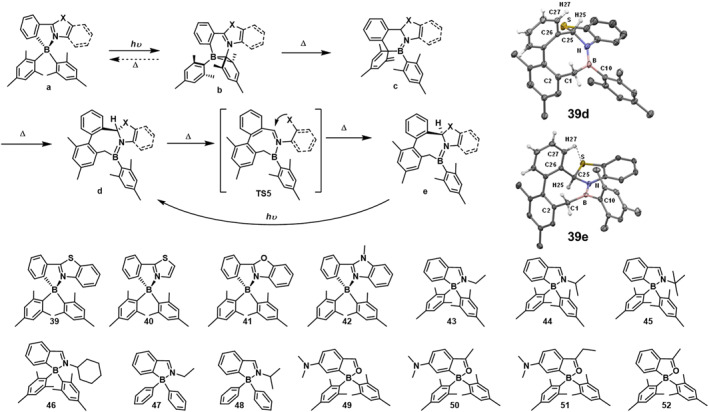
Photoisomerization and molecular structure of **39‐52**, Crystal structures of **39d** and **39e**.[^52^, ^53^, ^54^] Reproduced with permisson.^[52]^ Copyright 2013, American chemical society. Reproduced with permission.^[53]^ Copyright 2018, Royal Society of Chemistry. Reproduced with permission.^[54]^ Copyright 2019, American chemical society.

The compounds **39**‐**42**,^[52]^ obtained by replacing the pyridine moiety in PPy with azole, undergo unprecedented multiple structural transformations under illumination or heating, sequentially generating isomers **b**, **c**, **d**, and **e** (Figure 16). Initially, similar to **PPyBMes2**, they form a B‐C‐C three‐membered cyclic structure upon irradiation at 350 nm, exhibiting color changes to blue, deep blue, or purple. When heated, the dark isomer **b** undergoes **a** rare intramolecular hydrogen atom transfer (HAT) process, reducing the imidazole ring and giving rise to a new isomer **c**. This isomer c further transforms into isomer d, driven by the rearomatization of the cyclohexadienyl (CHD) ring in c, which induces a 1,3‐sigmatropic shift of the B atom. Consequently, the six‐membered ring in **c** expands into an eight‐membered ring in **d**. The high thermodynamic stability of isomer d and the relatively lower aromaticity of the azole ring compared to pyridine are believed to be the key driving forces for the c‐to‐d conversion in this system. During the **39d**‐to‐**39e** process, the C25 atom in the thiazole ring transitions from a sp^2^ to a chiral sp^3^ carbon, while the C1 methyl group bonded to an aliphatic C atom in **39b** transforms into a CH_2_ group, forming a *σ* bond with the B atom. A crucial distinction between **39d** and **39e** lies in the inversion of the chiral carbon atom C25 configuration. The boron unit plays a pivotal role in the reversible conversion between **39d** and **39e**. The reversible diastereomeric interconversion between **c** and **d** represents a rare phenomenon. These examples illustrate the chameleon‐like behavior of chelated boron compounds, exhibiting resilience in participating in/supporting complex molecular transformations in response to external stimuli such as light and heat without undergoing decomposition. The internal donor unit attached to the aryl ring is crucial for achieving the rich photo/thermochemical reactivity of boron compounds.

When the azole unit is replaced by an imine, compounds **43**‐**46** ^[53]^ also support multi‐structural transformations similar to those observed in compound **39**. Upon illumination, these compounds form their respective dark isomers, exhibiting a strong deep purple color. However, the purple color of **43**‐**46** persists for only a few seconds, and the solution returns to being colorless, indicating a short lifetime of the dark isomers. Continued illumination leads to the complete conversion of **b** to **c** and **c** to **d**. Additionally, compounds **47** and **48**, which do not have bulky aryl groups, also exhibit a photoreaction pathway similar to that of compound **43**. This may be due to the significantly reduced contribution of the benzyldiamine unit to the *π*‐π* of the S_1_ state compared to the diaryl chelating unit. This represents the first example of a photoreaction in N‐C chelated boron compounds with small aromatic rings, suggesting that simplified compounds are more reactive than the PPy‐chelated system, thereby expanding the reaction system of N‐C chelated boron compounds.

In 2019, Hu et al.^[54]^ reported a class of C‐O chelated boron compounds that exhibit a photoreaction process similar to that of compound **39**. Under a 365 nm lamp, compounds **49**‐**52** convert to their respective dark‐colored isomers **49a**‐**52a** at low temperatures. However, when the temperature exceeds −40°C, the molecular structure rapidly reverts to **49**‐**52**. Upon illumination at room temperature, compounds **49**‐**52** undergo a direct transformation into **49b**‐**52b**, further isomerizing into **49c**‐**52c**, forming eight‐membered ring‐expansion products similar to those observed in imine analogs.

### 4.2 The regulation of photoresponsive behavior in tetracoordinate arylboron compounds via modification of the aryl (Ar) connected to boron

**FIGURE 17:**
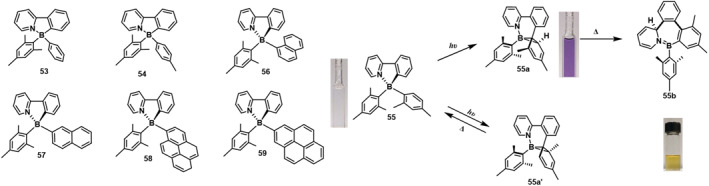
Molecular structure of **53‐59**, and photoisomerization of **55**. Reproduced with permission.^[55]^ Copyright 2017, John Wiley and Sons. Reproduced with permission.^[56]^ Copyright 2016, American Chemical Society.

In 2017, Mellerup et al.^[55]^ established the first asymmetric N,C‐chelated boron compound that undergoes regioselective photoisomerization and C‐C bond formation. This was done to deeply explore the impact of B‐substituent steric hindrance and symmetry on the photoisomerization of chelated boron compounds. Compounds **53** and **54**, which are similar to compound **32**, exhibit two consecutive color changes under UV lamp irradiation (300 or 365 nm). They transition from being colorless to bright pink and then to orange. This color change is attributed to the formation of isomer b upon initial irradiation, which subsequently converts to isomer c due to the heat generated from prolonged irradiation. The photoisomerization of B(PPy)(Mes)(Ar) shows regioselectivity towards both the smaller aryl ring and the less congested carbon atom when forming a new B‐C bond with the aryl ring. Compound **55**, containing a 2,4‐dimethylphenyl group, demonstrates a preference for forming isomer **55a** (70%) at the less sterically hindered position, while isomer **55a'** (30%) is formed at the more hindered position. Upon heating, **55a** transforms into a BN seven‐membered heterocyclic product **55b**, whereas **55a'** reverts back to **55** (Figure 17). They highlight the generality of this transformation and underscore the critical roles of both steric and electronic factors in this class of molecules. Furthermore, for the first time, direct hydrogen atom migration reactions involving the borirane ring of the dark isomers have been documented.

Replacing the Ar unit in B(PPy)(Mes)(Ar) with polycyclic aromatic hydrocarbon (PAH) rings, such as 1‐naphthyl, 2‐naphthyl, 1‐pyrenyl, and 2‐pyrenyl, does not alter the photoreaction pattern of these asymmetric compounds. Only boranes similar to **32c** are formed on the PAH substituents. Studies have shown that 2‐position substituted aromatic hydrocarbons can significantly improve their quantum yield. These molecules can form corresponding dark isomers under illumination. Compounds **56**‐**58** ^[56]^ undergo B‐C bond cleavage and form B‐C seven‐membered heterocyclic rings with the aromatic hydrocarbons upon heating. The high temperature required for this process may be due to the stabilization of the borane ring by the extended *π*‐conjugation of naphthalene. However, **59a** converts back to **59** upon heating, demonstrating photothermal reversibility. The very low ΦPI of **58** and **59** is attributed to the fact that the pyrenyl group has a triplet energy closest to that of the boron group, allowing them to act as triplet acceptors.

**FIGURE 18:**
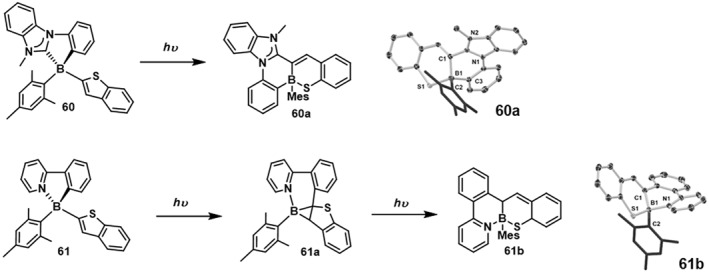
Photoisomerization of **60** and **61,** Crystal structures of **60a** and **61b**. Reproduced with permission.^[57]^ Copyright 2018, John Wiley and Sons.

In 2018, Mellerup et al.^[57]^ synthesized chiral chelated boron compounds **60** and **61** (Figure 18). Upon illumination, the minor product **61a** was detected, while the major product was the air‐stable **61b**. Compound **61b** represents the first example of an isoaromatic C‐S bond being activated by the main group system and is also a previously unknown 1,2‐benzothiazole purine derivative. The pyridine ring in **61** can be replaced by N‐methylbenzimidazole, and the resulting boron compound undergoes a similar phototransformation to **61**, forming an analog of **61b**. Interestingly, the photoisomerization of **60** did not yield the expected product **60b**, which might be due to C−C coupling between the phenyl and thiophene rings. Instead, the isomer compound **60a** was obtained quantitatively, with a C−C bond formed between NHC and the thiophene ring. These results provide a promising strategy for activating chemical bonds through reactive excited state intermediates.

Thus far, the chelating ligands in the described systems have played a pivotal role in various unprecedented phototransformations. Apart from providing highly directed CT transitions, which lead to the formation of necessary biradical species, the chelating ligands also exert a stabilizing effect on the boron core through these versatile structural transformations.

## 5 THE PHOTORESPONSIVE BEHAVIOR OF C‐C/N‐C CHELATED ARYLBORON COMPOUNDS: FROM B‐C‐C THREE‐MEMBERED CYCLIC STRUCTURE TO THE MIGRATION OF ARYL GROUPS

Upon illumination, tetra‐chelated boron compounds undergo the formation of B‐C‐C three‐membered cyclic structure, followed by ring expansion or the departure of aryl boron to generate new structures. Additionally, novel aryl migration phenomena can occur through changes in ligand structure, particularly in B‐N six‐membered heterocyclic core and N‐heterocyclic carbene‐based boron compounds, which serve as typical examples.

**FIGURE 19:**
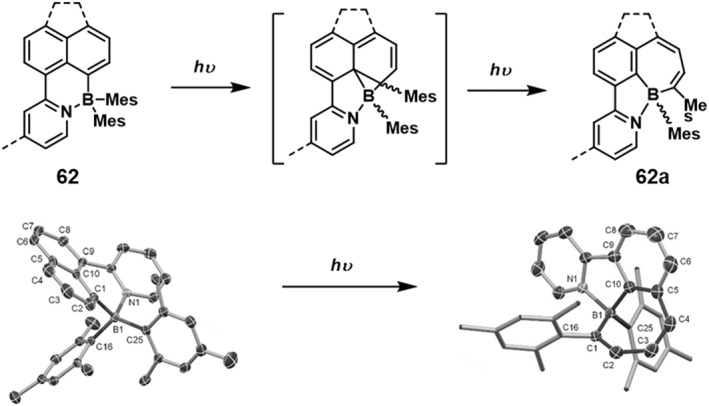
Photoisomerization of **62,** Crystal structures of **62** and **62a.** Reproduced with permission.^[58]^ Copyright 2018, John Wiley and Sons.

In 2017, Wang et al.^[58]^ designed compound **62**, featuring a 2‐pyridylnaphthalene ligand and a tetracoordinate arylboron compound (Figure 19). This system exhibited an unprecedented isomerization phenomenon. Upon irradiation with 350 nm light, the benzene ring connected to the boron atom within the naphthalene moiety was opened, resulting in the insertion of the boron atom into the benzene ring and the formation of a novel B‐hybrid seven‐membered ring. Concurrently, a migration of one of the mesityl groups on the boron atom occurred to the adjacent carbon atom, involving a new B‐C‐C three‐membered cyclic process during the reaction. Compound **62a** demonstrated air stability and could be purified through chromatographic methods. The crystal structure of **62a** was further elucidated via X‐ray single crystal diffraction analysis. The observation of such a rare phenomenon, where an aromatic ring is opened under light irradiation, constitutes a noteworthy exception in the field of photochemistry.

**FIGURE 20:**
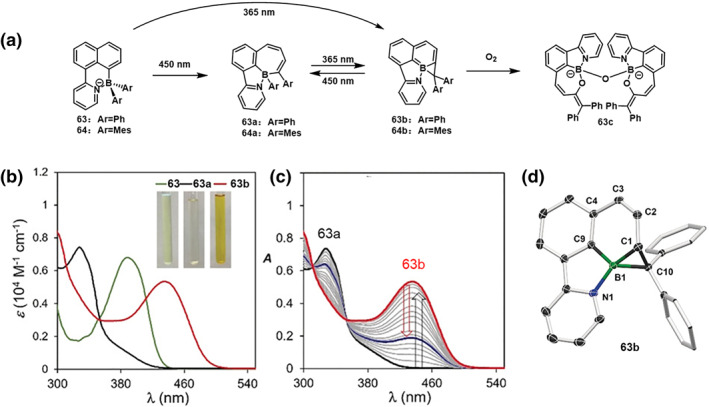
(a) Photoisomerization of **63** and **64** (b) UV/Vis spectra of **63**, **63a**, and **63b** in toluene and photographs showing their colors in benzene. (c) UV/Vis spectral tracking showing the full conversion of **63a** into **63b** at 365 nm and the partial conversion of **63b** into 63a at 450 nm. (d) Crystal structures of **63b.** Reproduced with permission.^[38]^ Copyright 2019, John Wiley and Sons.

In 2019, He et al.^[38]^ designed a naphthylpyridine ligand‐based compound **63**, aiming to investigate the impact of incorporating boron atoms from the N‐C chelated organoboron system into a fully conjugated six‐membered ring system. This compound exhibited complete isomerization when exposed to 365 nm irradiation, resulting in the quantitative formation of **63b**. The photoisomerization quantum yield for the conversion of **63** to **63b** was found to be 34%. Furthermore, irradiation of **63b** at 450 nm led to the emergence of **63a**. The interconversion between **63a** and **63b** using distinct wavelengths was attributed to their well‐separated absorption bands, with λmax values of 330 nm for **63a** and 440 nm for **63b** (Figure 20). It's worth noting that **63b** represents the first example of a boron‐containing tropone analog featuring bond rearrangement. Compound 63b demonstrated stability towards water but reacted with oxygen, undergoing a color change from orange to yellow to form **63c**. This new compound comprises two NCO‐chelated boron atoms and two oxaborepin rings, with the two boron units connected in an approximate *C*_*2*_ symmetry. Similar photoisomerization behavior was observed for compound **64**. Through TD‐DFT calculations, the critical role of electronic and steric factors in this unusual structural transformation was elucidated.

**FIGURE 21:**
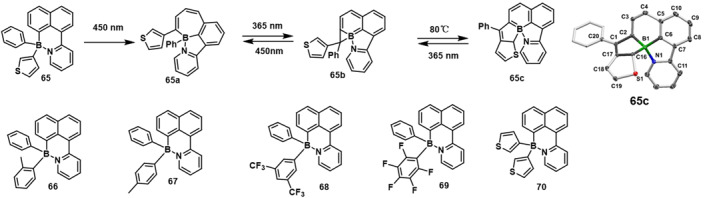
Molecular structure of **65‐70** and photoisomerization of **63**. Reproduced with permission.^[59]^ Copyright 2020, John Wiley and Sons.

Continuing their exploration, they further designed and synthesized a novel class of N,C‐chelated boron heterocyclic compounds. This new series included asymmetrically substituted boron molecules labeled 65‐70 (Figure 21).^[59]^ Under the influence of 450 nm illumination, all these compounds could transform into their corresponding dark isomers, denoted as **a**. Compound **65** exhibited remarkable regioselectivity, characterized by the migration of the smaller aromatic ring to produce isomer **a**. Through careful regioselective photoconversion, compound **65** was successfully converted into borepin **65a**. Subsequently, borepin **65a** underwent photoisomerization to yield the diastereomeric pair **65b** RR/RS. Upon heating the mixture, a distinct isomer **c** was formed. This isomer exhibited the unique ability to revert back to either **b** or **a** upon exposure to different wavelengths of light. During the entire illumination process, the borane molecules displayed a reversible rearrangement between boracycloproane and boracyclopentane configurations. Compound **70** also showcased similar photoresponsive behavior. However, it was observed that compounds **66‐69** were capable of forming only isomer **b**. This groundbreaking research underscores the profound richness and complexity inherent in the photochemistry and photoconversion of boron systems. Moreover, it opens up new avenues for the discovery of novel chemistries and species within the realm of organoboron compounds.

**FIGURE 22:**
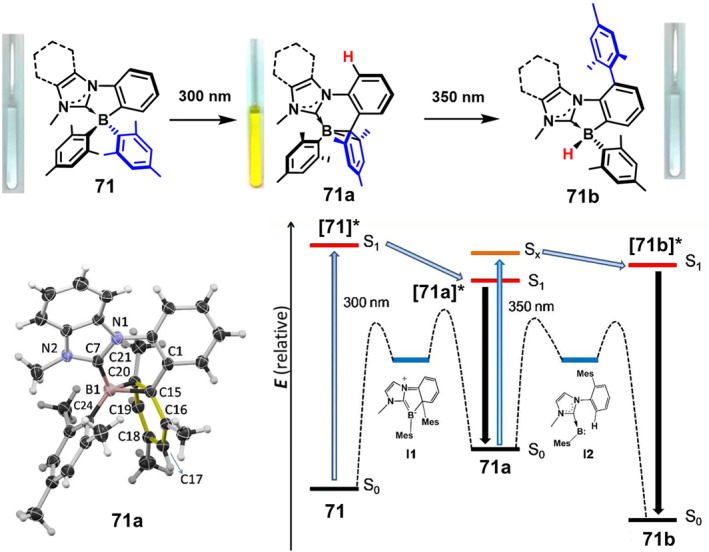
Photoisomerization of **71** and Crystal structures of **71a**, Energy diagrams showing the relative energies of **71**, **71a**, and **71b** at the ground state (S_0_) and at the first excited state (S_1_). Reproduced with permission.^[60]^ Copyright 2012, American chemical society.

In 2012, Rao et al.^[60]^ designed a C‐C chelated boron compound **71** that exhibits unprecedented structural transformations. Upon excitation at 300 nm, compound **71** changes color from colorless to bright yellow. Additionally, when irradiated at 350 nm, **71a** gradually loses its color and converts into a new colorless isomer **71b** (Figure 22). This two‐stage photoconversion phenomenon is attributed to the strong electron‐donating ability of the N‐heterocyclic carbene (NHC) ligand, which stabilizes various intermediates and transition states during the conversion process. Theoretical calculations indicate that the transition from [**71**]* to [**71a**]* is an exothermic process with an energy difference of approximately 50 kJ·mol^−1^. Therefore, the conversion of **71** to **71a** can occur easily through the excited state. In the first excited state, [**71b**]* has an energy approximately 30 kJ mol^−1^ higher than that of [**71a**]. Thus, to convert **71a** to **71b**, excitation of [**71a**] at 350 nm is required. The ability of C, C‐chelated BMes_2_ compounds to undergo stepwise photoisomerization is remarkable, opening up numerous new opportunities to obtain unusual structures through excited states.

## 6 OTHER PHOTORESPONSIVE BEHAVIORS OF TETRACOORDINATE ARYLBORON COMPOUNDS

In addition to the common N‐C chelating tetracoordinate arylboron compounds, O‐C chelating boron compounds and others also exhibit unique photoresponse mechanisms distinct from those mentioned above. These molecules will be described in detail in this section.

**FIGURE 23:**
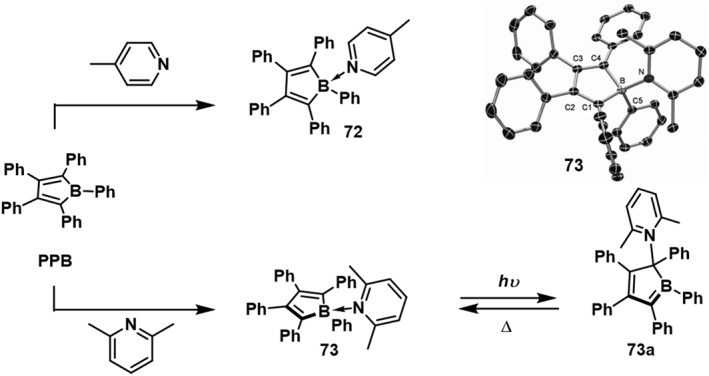
Syntheses of the Lewis base adducts **72** and **73**, and the light‐induced rearrangement of **73**. Reproduced with permission.^[61]^ Copyright 2011, John Wiley and Sons.

In 2011, Braunschweig et al.^[61]^ observed that treating a CDCl_2_ solution of 1,2,3,4,5‐pentaphenyl‐1*H*‐borole (**PPB**) with 4‐methylpyridine immediately changed its color from blue to yellow (Figure 23). However, when 2,6‐dimethylpyridine was added to **PPB**, the solution retained its deep blue color. Upon cooling to 40°C, the color shifted from deep blue to bright yellow. This color change is attributed to the fact that at room temperature, approximately 29% of **PPB** remains uncoordinated, preserving the characteristic blue color of the **PPB** boron ring. When toluene solutions of **72** and **73** were illuminated at 50°C, no significant change was observed for **72**. However, for compound **73**, a color change from yellow to dark green was noticed. This change is due to the transfer of a pyridine analog from the boron atom to an adjacent carbon atom, forming a B‐C bond. In the absence of light, compound **73a** completely converts back to **73** at room temperature without undergoing decomposition.

**FIGURE 24:**
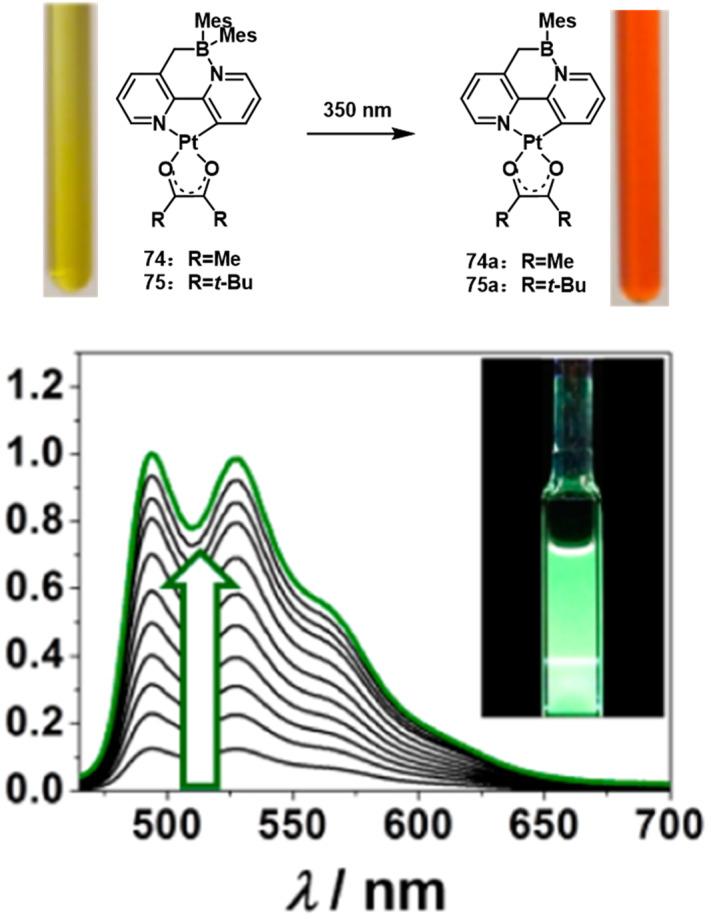
Emission spectra showing the conversion of **74** and **75** in toluene to their corresponding benzoquinoline compounds upon irradiation at 350 nm. Reproduced with permission.^[62]^ Copyright 2014, American chemical society.

In 2014, Ko et al.^[62]^ synthesized a class of Pt(II)‐functionalized 1,2‐azaborines, denoted as **74** and **75** (Figure 24). When excited at 350 nm, these compounds underwent a mesityl photoelimination reaction, resulting in a color change of their solutions from yellow to orange‐red. The study revealed that metal chelation and internal hydrogen bonding significantly enhance the photoelimination quantum yield of B, N‐heterocycles by lowering the activation barrier and reducing the excitation energy. This research demonstrates that utilizing metal chelation/coordination to manipulate photochromic systems is an effective approach to boost the quantum yield of photochemical reactions.

**FIGURE 25:**
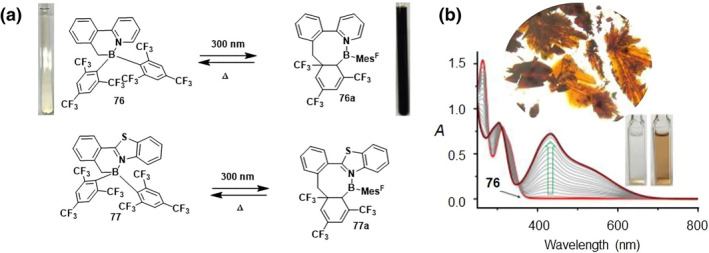
(a) Scheme showing the interconversion of **76/77** and **76a/77a**. (b) UV−vis spectra showing the conversion of **76** to **76a** in THF and photographs showing the crystals of **76a** and solution color of **76** and **76a**. Reproduced with permission.^[39]^ Copyright 2016, American chemical society.

The highly electron‐deficient mesityl with trifluoromethyl substitution of B, N heterocycles **76** and **77** exhibit another novel photoisomerization phenomenon.^[39]^ Under irradiation, these compounds do not undergo elimination reactions but instead rapidly convert to **76a** through a process of stereoselective and quantitative isomerization (Figure 25). In the structure of **76a**, the CH_2_ group has shifted its bonding from the boron atom to form a C−C bond with a CF_3_‐substituted quaternary carbon atom within the Mes^F^ ring, resulting in a distinctive eight‐membered ring structure. It's worth noting that this transformation is thermally reversible, and both molecules maintain their integrity even after undergoing 13 cycles of photo‐thermal conversion. Upon exposure to 300 nm irradiation, compound **77** undergoes a photoswitching process similar to that of **76**, yielding a dark brown isomer, **77a**. This observation suggests that compounds **76** and **77** constitute a new family of photochromic organic boron compounds.

**FIGURE 26:**
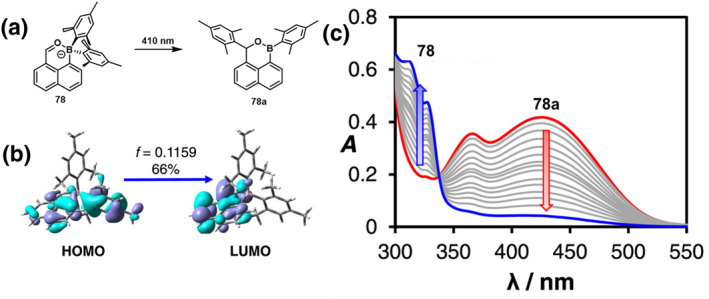
(a) Structural changes before and after 410 nm irradiation of **78.** (b) The diagrams of key orbitals of **78** involved in vertical excitations to S_1_. (c) Absorption spectral change of **78** in toluene upon 410 nm irradiation. Reproduced with permission.^[63]^ Copyright 2023, American Chemical Society.

In 2023, Zhou et al.^[63]^ designed a class of C‐O chelating boron compounds denoted as **78** (Figure 26), utilizing naphtha‐aldehyde as the ligand. Under 410 nm light irradiation, the solution's color shifted from light orange to light yellow. This color change was preceded by the weakening of the B‐Araxial bond within compound **78**, followed by the migration of the Ar group to the C atom bonded to the O atom via a 1,3 sigmatropic mechanism. Compound **78** demonstrated not only photoresponsiveness but also thermal reactivity. Upon heating at 60°C for a specified duration, all compounds underwent quantitative conversion to the same isomerization products that were previously obtained through photochemical pathways. The boron center's coordination with the O atom of the carbonyl group augmented the electrophilicity of the adjacent C atom. Simultaneously, the formation of the B‐O bond led to the attenuation of the B‐Ar bond, thereby facilitating the Ar group's migration towards the carbonyl carbon atom through a nucleophilic addition pathway, ultimately yielding Ar migration products.

## 7 CONCLUSION

Photoresponsive tetracoordinate arylboron compounds constitute a significant molecular system in the construction of photoresponsive materials, owing to their intriguing photochemical mechanisms and photophysical transformations. This class of molecular systems has undergone rapid development in recent years, giving rise to numerous novel organic boron photoresponsive molecular systems. Herein, we present a review of the recent advancements in this domain, categorized based on distinct molecular structures and photoisomerization mechanisms.

Majority of these arylboron compounds undergo the formation of a B‐C‐C three‐membered cyclic structure during their photoresponse. Certain molecules exhibit excellent photothermal reversibility, making them suitable candidates for applications in photochromic materials, molecular switches, and optical storage. Following the formation of the B‐C‐C ring, some molecules can further undergo intramolecular ring expansion or aryl migration upon exposure to light or heat. This versatility not only enables these molecules to be utilized in the aforementioned applications but also endows them with high synthetic value for the preparation of specialized compounds or materials. Additionally, this article summarizes various other photoinduced isomerization processes, providing deeper insights into the photoresponse behavior of this series of molecular systems.

When designing photoresponsive tetracoordinate boron aryl molecules, several key considerations must be taken into account: (1) In N‐C chelating molecules, the Ar group should be spatially bulky, such as mesityl, while avoiding fluoro‐substituted aromatic rings. (2) It is imperative to minimize the conjugation extension on the ligand. (3) The formation of a six‐membered heterocyclic system between boron and ligands can exhibit isomerization behavior distinct from that of **PPyBMes**_**2**_. (4) The influence of metal ions on the **PPyBMes**_**2**_ system warrants further investigation through the alteration of metal ion positions and binding sites, potentially unveiling novel and intriguing photochemical reactions.

Despite witnessing significant progress in the development of photoresponsive tetracoordinate boron aryl molecular systems in recent years, numerous challenges still lie ahead. Primarily, there is considerable scope for improvement in the reversibility and air stability of these molecules' photoresponse, which remains a critical hurdle for their practical application. Consequently, further refinement and optimization of molecular structures are essential. Secondly, the utilization of these molecules in optoelectronic devices remains limited, necessitating additional research and development efforts. Thirdly, the exploration of intelligent composite materials based on these molecules is still in its infancy, presenting opportunities for the targeted development of stable photochromic glass, optical storage media, wearable devices and so on.

Fortunately, with the rapid advancement of technologies such as molecular design, synthesis, characterization, and theoretical calculations, the discovery of additional photoresponsive tetracoordinate boron aryl molecular systems is on the horizon. As these challenges are gradually overcome, this important class of photoresponsive materials is poised to experience accelerated development. Through interdisciplinary collaboration and cooperation, these materials will increasingly find their way into our daily lives, driving social and economic progress.

## CONFLICT OF INTEREST STATEMENT

The authors declare no conflicts of interest.

## Data Availability

The data that support the findings of this study are available from the corresponding author upon reasonable request.
